# Sagittal plane alignment evaluation during walking in adult spinal deformity: gait analysis using DARTFISH

**DOI:** 10.1186/1748-7161-10-S1-P21

**Published:** 2015-01-19

**Authors:** Junya Katayanagi, Takahiro Iida, Yasumasa Oyama, Akihisa Ato, Ken Mine, Takashi Toumura, Satoru Ozeki

**Affiliations:** 1 Dokkyo Medical University Koshigaya Hospital, Japan

## Background

There are many evaluation tools of spinal deformities such as sagittal vertical axis (SVA), lumbar lordosis (LL), thoracic kyphosis (TK), pelvic tilt (PT), pelvic incidence (PI), sacral slope (SS) and so on. But no reliable parameters have been defined for measurements of spinal alignment during gait analysis.

## Objective

The purpose of this study is to assess the validity of our EGA-DARTFISH kinetic analysis parameter against established x-ray parameters and to evaluate its viability as an alternative to SVA.

## Material and method

**Figure 1:**
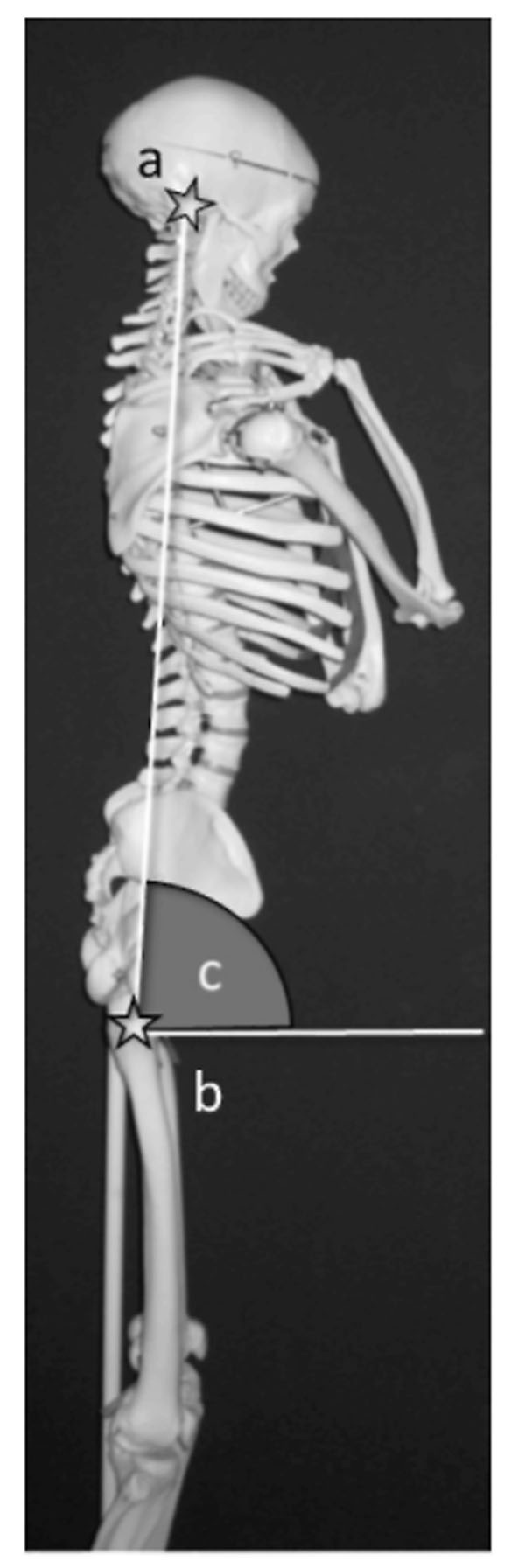
a: External auditory pore, b: Greater trochanter, c: “EGA”

**Figure 2:**
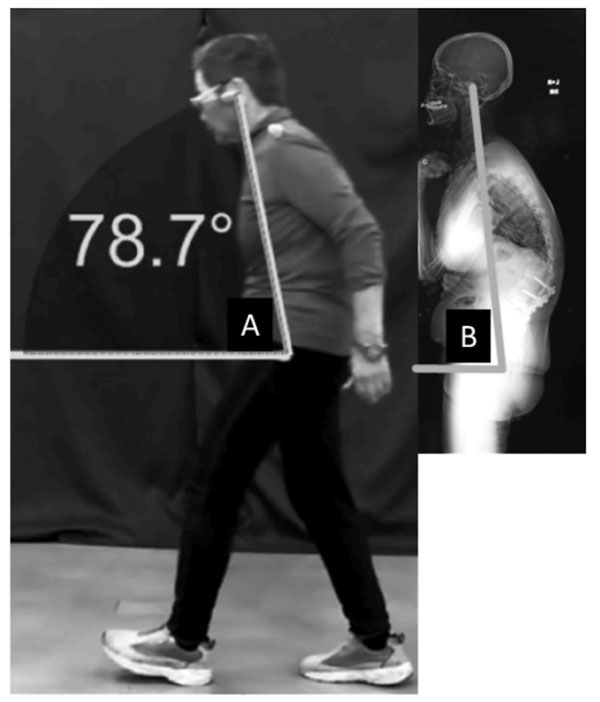
A: “EGA-DARTFISH” B: “EGA-DARTFISH”

We researched consequent 40 adult spinal deformity patients in our institute during January 2012 to January 2013. The mean age of patients was 60.1 years old. The research item was SVA, LL, TK, PT, PI, SS and EGA. We have movie recording from the side of the patients walking. We set markers at the External auditory pore and the Greater trochanter, and de fine the straight line between them as the EG line. We then measure the angle between the horizontal and EG lines. We call the angle “EGA-DARTFISH” if measured (over time) during gait analysis, and “EGA-XP” if measured radiographically. [Fig. 1, 2] We used the StatFlex ver.6.0 the statistical processing. We were calibrated in order to perform more accurate measurement. In this measurement, distortion at the screen edges is negligible.

## Result

**Figure 3:**
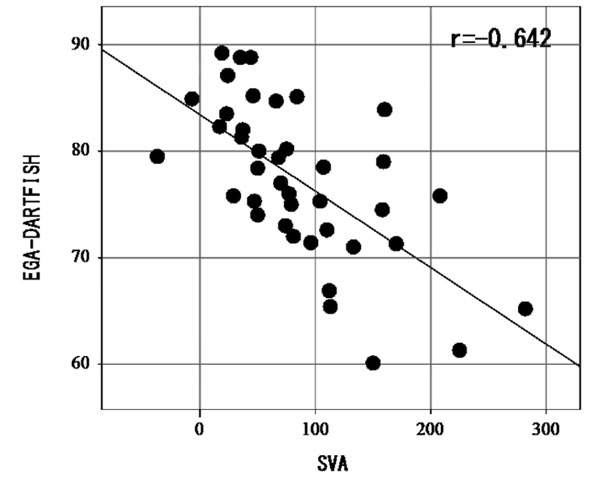
SVA ≤ 50mm group, r = -0.007 (no correlation), 50 < SVA ≤ 150mm group, r = -0.813 (strong negative correlation)

**Figure 4:**
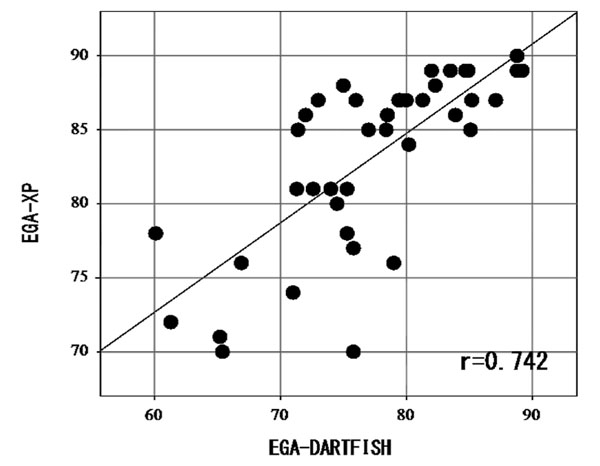
r = 0.727 moderate positive correlation

The average of various parameters were SVA = 88.2mm, EGA-XP = 85 degrees, EGA-DARTFISH = 77.7 degrees. There was light negative correlation between SVA and EGA (r=-0.642). There was no correlation in the group of less than 50mm SVA (-0.007), on the other hand there was strong negative correlation in the group of 50mm to 150mm SVA (-0.813) [Fig. 3]. There was strong positive correlation between EGA-DARTFISH and EGA-XP (r=0.742) [Fig. 4]. The difference between EGA-XP and EGA-DARTFISH was 6.05 degrees. This study suggested that patients would stoop of about 6 degrees during walking.

## Conclusion

Gait analysis using EGA-DARTFISH provided good visibility and reproducibility for measuring spinal sagittal alignment. In adult spinal deformity, the anteversion angle while walking is 6 degrees. EGA DARTFISH may be an appropriate alternative to SVA for measuring sagittal alignment.

## Consent

Written informed consent was obtained from the patient for the image (s) used in this study. A copy of the written consent is available for review by the Editor of this journal.

